# Author Correction: Is negative capacitance FET a steep-slope logic switch?

**DOI:** 10.1038/s41467-020-14795-y

**Published:** 2020-02-24

**Authors:** Wei Cao, Kaustav Banerjee

**Affiliations:** Department of Electrical and Computer Engineering, University of California, Santa Barbara, CA 93106 USA

**Keywords:** Electronic devices, Electronic devices, Electronic and spintronic devices

Correction to: *Nature Communications* 10.1038/s41467-019-13797-9, published online 10 January 2020.

The original version of this Article contained an error in the Peer Review file, which incorrectly omitted the last page. This has been corrected in both the PDF and HTML versions of the Article.

