# Supplementary material for: Is negative capacitance FET a steep-slope logic switch?
Source: Nat Commun. 2020 Jan 10;11:196. doi: 10.1038/s41467-019-13797-9 (PMC6954266; doi:10.1038/s41467-019-13797-9)
Supplement: Supplementary file 1 — Supporting Info [file 41467_2019_13797_MOESM1_ESM.pdf]

Supplementary Information for

Is negative capacitance FET a steep-slope logic switch?

Wei Cao and Kaustav Banerjee*

Department of Electrical and Computer Engineering

University of California, Santa Barbara, CA 93106

*correspondence to: kaustav@ece.ucsb.edu

Supplementary Note 1: Steep-Slope Devices

Steep slope, or small subthreshold swing (*SS*) can help field-effect transistors (FETs) save supply voltage (V_{dd}) and hence dynamic (switching) power that has a quadratic dependence on the V_{dd}, with fixed on-current (I_{on}) and off-current (I_{off}), as illustrated in **Supplementary Figure 1a**.

Supplementary Figure 1b summarizes several steep-slope device concepts as well as their pros and cons. Among these steep-slope devices, negative capacitance- (NC-) FETs have been attracting most research efforts of both academia and industry.

Supplementary Table 1 lists the most important metrics of several recently reported "NC-FETs". It can be found that steep slopes are generally observable only at low current level. Moreover, hysteresis begins to show up under alternating current (AC) condition even below MHz frequency. Such problems are not desired in logic application, and should not happen in a properly designed genuine NC-FET.

Supplementary Note 2: Quasi-Static Polarization State in Ferroelectric System

According to Landau-Devonshire theory, the Gibbs free energy density (U) of a single-domain ferroelectric (FE) material is given by,

$$U = \alpha P^2 + \beta P^4 + \gamma P^6 - E \cdot P \qquad \text{(S2a)}$$

where P is polarization, E is electric field. **Supplementary Figure 2a** shows the Gibbs free energy landscape under different electric field. The circles represent stable (valley regions) and metastable (barrier region, indicated with green shade) P states for different values of E. By letting $dU/dP = 0$, a continuous P-E curve can be obtained,

$$E = 2\alpha P + 4\beta P^3 + 6\gamma P^5 \qquad \text{(S2b)}$$

which includes all the possible polarization states of a FE material in response to an external electric field. In this curve, there is a portion exhibiting negative P-E slope (**Supplementary Figure 2b**), which, in physics, represents a negative capacitance (NC) region[2]. The NC region corresponds to the barrier region in the energy landscape (**Supplementary Figure 2a**), which is metastable in an isolated FE system. It is

worthwhile to note that the absolute value of the *P-E* slope is generally very large, i.e., the absolute NC dielectric constant $|\varepsilon_{NC}|$ is very large. For example, $|\varepsilon_{NC}|$ of the CMOS compatible $Hf_{0.5}Zr_{0.5}O_2$, can reach ~ $75\varepsilon_0$, which is more than three times that of HfO_2. It was predicted that by connecting a FE in series with a normal dielectric (DE) (**Supplementary Figure 2c**), with proper capacitance matching between FE and DE, i.e., $|C_{FE}| \geq C_{DE}$, the stack energy can be stable[2], as schematically shown in **Supplementary Figure 2d**.

Supplementary Note 3: Detailed Derivations of Equations in Figures 2 and 4

The drain current of a (n-type) FET can be expressed as

$$I_d = W \cdot v \cdot Q_e \qquad \text{(S3a)}$$

where *W* is device width, and *v* is carrier velocity, Q_e is the electron charge density in the channel,

$$Q_e = q \cdot \sum_i \int_{E_{c,i}}^{+\infty} \frac{DOS_i}{1 + exp(\frac{E - E_f}{kT})} dE$$

$$= q \cdot kT \cdot \sum_i DOS_i \cdot ln\left(1 + e^{\frac{E_f - E_{c,i}}{kT}}\right) \qquad \text{(S3b)}$$

All other symbols in this equation have the same meaning as that in Equation 2 in the main manuscript, and have been explained there. Note that quantum capacitance C_Q is the first derivative of Q_e w.r.t the channel potential and can be obtained from Equation 2 in the main manuscript.

The slope of the I_d-V_g curve of a NC-FET is

$$S_{NC-FET} = \left(\frac{d[logI_d]}{dV_g}\right)^{-1} = \frac{dV_{MOS}}{d\varphi_{Ch}} \cdot \frac{dV_g}{dV_{MOS}} \cdot \frac{d\varphi_{Ch}}{d[logI_d]}$$

Where V_g is the gate voltage, V_{MOS} is the voltage at the interface of NC and oxide, and φ_{ch} is the channel potential, as illustrated in **Figure 1** in the main manuscript. According to the small-signal capacitor network shown in **Figure 1b** in the main manuscript, $(\Delta V_{MOS} - \Delta\varphi_{Ch})\cdot C_{OX} = \Delta\varphi_{Ch}\cdot C_{div}$, and $(\Delta V_g - \Delta V_{MOS})\cdot C_{NC} = -(\Delta V_g - \Delta V_{MOS})\cdot|C_{NC}| = \Delta V_{MOS}\cdot C_{MOS}$, above equation can be converted to

$$S_{NC-FET} = \left(1 + \frac{C_{div}}{C_{OX}}\right) \cdot \left(1 - \frac{C_{MOS}}{|C_{NC}|}\right) \cdot \frac{I_d d\varphi_{Ch}}{dI_d} \cdot ln10 \qquad \text{(S3c)}$$

Substituting Equation 3 in the main manuscript and **Equation S3a** into above equation, and considering the lowest conduction mode (i.e., i=1, $DOS=DOS_1$, $E_c=E_{c,1}$) only,

$$S_{NC-FET} = \left(1+\frac{C_{div}}{C_{OX}}\right)\cdot\left(1-\frac{1}{|C_{NC}|}\frac{C_{div}C_{OX}}{C_{div}+C_{OX}}\right)\cdot\frac{q\cdot kT\cdot DOS\cdot ln\left(1+e^{\frac{E_f-E_c}{kT}}\right)d(-E_c/q)}{q\cdot kT\cdot DOS\cdot dln\left(1+e^{\frac{E_f-E_c}{kT}}\right)}\cdot ln10$$

$$=\left(1-\frac{1}{|C_{NC}|}\frac{C_{div}C_{OX}}{C_{div}+C_{OX}}+\frac{C_{div}}{C_{OX}}-\frac{C_{div}}{C_{OX}}\frac{1}{|C_{NC}|}\frac{C_{div}C_{OX}}{C_{div}+C_{OX}}\right)\cdot\frac{\left(1+e^{\frac{E_f-E_c}{kT}}\right)ln\left(1+e^{\frac{E_f-E_c}{kT}}\right)}{e^{\frac{E_f-E_c}{kT}}}\cdot\frac{kT}{q}\cdot ln10$$

$$=\left(1-C_{div}\left[\frac{1}{|C_{NC}|}-\frac{1}{C_{OX}}\right]\right)\cdot S_{TP} \qquad \text{(S3d)}$$

where

$$S_{TP}=\left(1+e^{\frac{E_c-E_f}{kT}}\right)\cdot ln\left(1+e^{\frac{E_f-E_c}{kT}}\right)\cdot 60$$

at room temperature. Note that in the subthreshold regime in which $E_f << E_c$, S_{TP} is reduced to

$$S_{TP}\approx e^{\frac{E_c-E_f}{kT}}\cdot e^{\frac{E_f-E_c}{kT}}\cdot 60$$

$$\approx 60\ mV\ per\ decade$$

which stems from the thermionic emission transport mechanism.

As have been discussed in the main manuscript, in the near and above- threshold regimes, C_Q dominates C_{div}, i.e., $C_{div}\approx C_Q$, **Equation S3d** becomes

$$S_{NC-FET}=\left(1-C_Q\left[\frac{1}{|C_{NC}|}-\frac{1}{C_{OX}}\right]\right)\cdot S_{TP}$$

As have been discussed in the main manuscript, in the subthreshold regime of a poorly designed short-channel NC-FET, short-channel capacitance C_{SCE}, which is mainly composed of source/drain geometry capacitance $C_{s/d,geo}$, dominates C_{div}, i.e., $C_{div}\approx C_{SCE}$, S_{TP}=60, **Equation S3d** is reduced to

$$SS_{NC-FET}=\left(1-C_{SCE}\left[\frac{1}{|C_{NC}|}-\frac{1}{C_{OX}}\right]\right)\cdot 60$$

For MOSFETs in which NC layer is absent, the slope of the I_d-V_g curve can be derived by making $|C_{NC}|$ infinitely large in the above equations,

$$S_{MOSFET} = \left(\frac{d[logI_d]}{dV_g}\right)^{-1}$$

$$= \frac{dV_g}{d\varphi_{Ch}} \cdot \frac{d\varphi_{Ch}}{d[logI_d]}$$

$$= \left(1 + \frac{C_{div}}{C_{OX}}\right) \cdot S_{TP} \qquad \text{(S3e)}$$

where

$$S_{TP} = 60 \cdot \left(1 + e^{\frac{E_c - E_f}{kT}}\right) \cdot ln\left(1 + e^{\frac{E_f - E_c}{kT}}\right)$$

In the near- and above- threshold regimes, $C_{div} \approx C_Q$, then,

$$S_{MOSFET} = \left(1 + \frac{C_Q}{C_{OX}}\right) \cdot S_{TP}$$

In the subthreshold regime of a short-channel MOSFET, $C_{div} \approx C_{SCE}$, S_{TP}=60, **Equation S3e** is reduced to

$$SS_{MOSFET} = \left(1 + \frac{C_{SCE}}{C_{OX}}\right) \cdot 60$$

Supplementary Note 4: Minimized $C_{MOS,<Vth}$ in Modern MOSFETs

Modern MOSFETs have evolved into the ultrathin-body (UTB) era, in the form of semiconductor-on-insulator (SOI)-FET, Fin-FET, nanowire (NW)-FET, carbon-nanotube (CNT)-FET, and 2D-FET et al. Active experimentalists have often flirted with NC on these "good" FETs, expecting to produce a device with combined inheritance of good electrostatics and NC effect[11,12]. However, the merits of these modern/good FETs make the voltage divider capacitance $C_{div,<Vth}$ (=C_{trap}+C_{dep}+$C_{s,geo}$+$C_{d,geo}$) negligibly small (**Supplementary Figure 3a**), thereby limiting their capability to benefit from NC. Also, many researchers may intuitively arrive at the conclusion that the UTB of modern MOSFETs can serve as

a large channel capacitance or a large effective C_{dep} (**Figure 1** in the main manuscript) which, however, is a fundamental misunderstanding of FET physics. In UTB MOSFET, body terminal is absent, i.e., the other side (w.r.t. the gate) of UTB is electrically floating. Therefore, the thickness of UTB does not determine the channel capacitance or effective C_{dep}. In fact, UTB suppresses $C_{div,<Vth}$ and hence $C_{MOS,<Vth}$ (=C_{OX} $C_{div,<Vth}$/(C_{OX}+$C_{div,<Vth}$)). Numerical simulation, by solving Poisson's and drift-diffusion equations in the entire device region, is performed to obtain the total gate capacitance C_{MOS} of a Si SOI MOSFET (see the inset in **Supplementary Figure 3b**) versus gate bias V_{MOS}. The doping density in the channel is set to be ~1×10^{16} cm^{-3}. As shown in **Supplementary Figure 3b**, C_{MOS} of a trap-free device in the subthreshold regime (-0.9V – -0.2 V) is negligibly small, w.r.t. gate oxide capacitance C_{OX}. As a result, it is hard to achieve sub-60 *SS*, unless unfeasibly thick NC layer is used, as illustrated in **Figure 2c** in the main text. From an NC operation point of view, subthreshold charge density $Q_{MOS,<Vth}$ (=$C_{MOS,<Vth} \cdot V_g$) in these "good" FETs induces (through electric displacement field D (=$C_{MOS,<Vth} \cdot V_g$), which is continuous across oxide/NC interface) negligible polarization in NC, and thus miniscule voltage gain A_V(=$|C_{NC}|$/($|C_{NC}|$-$C_{MOS,<Vth}$)) (Equation 4 in the main manuscript). Therefore, contrary to MOSFET design, a voltage divider (i.e., finite $C_{div,<Vth}$) should be intentionally introduced into NC-FETs to exploit A_v benefits. It is worthwhile to note that a mid-gap trap density of ~1×10^{11} cm^{-2} can introduce appreciable capacitance into $C_{MOS,<Vth}$. In other words, NC benefit and steep slope may be more readily observable in devices in which defective materials such as 2D, organic, amorphous and polycrystalline materials, and poor interfaces such as III-V/Oxide and Ge/Oxide are involved, compared to high-quality Si devices.

Supplementary Note 5: Large Quantum Capacitance in MOSFETs

In FETs, quantum capacitance C_Q describes the change of the mobile charges in response to channel potential modulation (Equation 2 in the main text). As shown in the simulation results in **Supplementary Figure 4**, even in the ultrathin channel of a 5 nm UTB SOI MOSFET with EOT of 1 nm, C_Q is still much larger than C_{OX}.

Supplementary Note 6: Derivation of Minimum *SS*

As indicated in **Figure 2d** in the main manuscript, minimum *SS* (SS_{min}) that does not suffer from hysteresis can be obtained by matching $|C_{NC}|$ to $C_{MOS,>Vth}$, i.e., by substituting $|C_{NC}| = C_{MOS,>Vth}$ into the *SS* formula in **Figure 2b** in the main manuscript,

$$SS_{min} = \left(1 - C_{div,<Vth}\left[\frac{1}{|C_{NC}|} - \frac{1}{C_{OX}}\right]\right) \cdot 60$$

$$= \left(1 - C_{div,<Vth}\left[\frac{1}{C_{MOS,>Vth}} - \frac{1}{C_{OX}}\right]\right) \cdot 60$$

According to Equation 3 in the main manuscript, $C_{MOS,>Vth}= C_{div,>Vth}\cdot C_{OX}/(C_{div,>Vth}+ C_{OX})$, then

$$SS_{min} = \left(1 - C_{div,<Vth}\left[\frac{1}{C_{div,>Vth}} + \frac{1}{C_{OX}} - \frac{1}{C_{OX}}\right]\right) \cdot 60$$

$$= \left(1 - \frac{C_{div,<Vth}}{C_{div,>Vth}}\right) \cdot 60$$

Substituting $C_{div,>Vth}= C_{div,<Vth} + C_Q$ into above equation, we arrive at

$$SS_{min} = \frac{C_Q}{C_Q + C_{div,<Vth}} \cdot 60$$

Supplementary Note 7: The Effect of NC Non-Linearity

The linear term (with coefficient α) in **Equation S2b**, gives a constant (bias independent) $\varepsilon_{NC}=1/(dE/dP)+\varepsilon_0=1/(2\alpha)+\varepsilon_0$ (and hence C_{NC}) as the 0th order approximation (**Supplementary Figure 5a**), which is valid in low-to-medium electric-field range, as indicated in **Supplementary Figure 2b**. Note that α is negative, which is the source of negative capacitance. When electric field approaches the coercive field of FE (the two turning points in **Supplementary Figure 2b**), the nonlinearity terms (with β and γ coefficients) begin to deviate the *P-E* relation from linearity.

To simplify the analysis, γ coefficient is set to be zero in this work. As a result, $|\varepsilon_{NC}|$ (and hence $|C_{NC}|$) increases with bias or charge density, as indicated by the derived formula in **Supplementary Figure 5a**. This non-linearity introduces a damping term to the slopes of both *SS*/60 and S/S_{TP} lines versus NC layer thickness T_{NC} (**Supplementary Figure 5b**), which can be depicted as a counterclockwise rotation of both S/S_{TP} and *SS*/60 lines, leading to enlarged NC design space, at the expense of increased *SS*, as illustrated

in **Supplementary Figure 5b**. **Supplementary Figures 5c** and **5d** show simulated I_d-V_g and S-I_d characteristics of a Si SOI NC-FET (inset), respectively, with different non-linearity term β. With increased β, hysteresis is effectively eliminated, at the expense of increased SS, which is consistent with the uncovered role of NC non-linearity in **Supplementary Figure 5b**. Note that the simulation here is performed on a fixed device structure/size. With larger β, NC design space is wider, SS_{min} can be lower if the device structure/size is optimized.

Note that although the effect of NC non-linearity on the design space of hysteresis-free SS has been discussed in a previous paper[13], the clear physics picture developed in this work allows us to explain this matter much more effectively, in terms of clarity, depth, and generality.

It is worth mentioning that the appearance of multi-domains and/or poly-crystals, w.r.t the ideal single-domain case considered in this work, in the ferroelectric layer may introduce additional and unpredictable non-linearity, because these discrete domains/crystals are unlikely to have uniform properties (P-E curves). As well known, today's scaled transistors already get severely loaded by 'bias-dependent' capacitances such as gate-to-source/drain capacitances $C_{g,s/d}$ and Miller capacitance etc, which makes the device/circuit behavior difficult to be precisely evaluated. The multi-domain and poly-crystalline ferroelectric layer added in NC-FETs, not only further complicate the device behavior, but also serve as a major source of performance variation in short-channel NC-FETs that have comparable feature sizes w.r.t. these domains/crystals.

Supplementary Note 8: The Mechanism of Internal Metal Gate (IMG) Aided NC-FET

An internal metal gate (IMG) inserted between the NC layer and oxide has been found able to achieve small SS in short-channel FinFETs, without introducing hysteresis[14]. This section clarifies the underlying physics using the same analysis developed in the main manuscript. **Supplementary Figure 6a** shows the capacitor network in a NC-FET with IMG. It can be found that two additional fringing (overlapping included) capacitors, $C_{frin,s/d\text{-}IMG}$, appear between source/drain and the floating IMG. S and SS formula are derived and revisited for this structure, as shown in **Supplementary Figure 6b**.

Note that the essential difference is that the additional $C_{frin,s/d\text{-}IMG}$ is in parallel with C_{MOS}, thus **Equation S3c** should be modified to be

$$S_{NC-FET} = \left(1 + \frac{C_{div}}{C_{OX}}\right) \cdot \left(1 - \frac{C_{MOS}+C_{frin,s/d-IMG}}{|C_{NC}|}\right) \cdot \frac{I_d d\varphi_{Ch}}{dI_d} \cdot ln10$$

$$= \left(1 + \frac{C_{div}}{C_{OX}}\right) \cdot \left(1 - \frac{C_{MOS}+C_{frin,s/d-IMG}}{|C_{NC}|}\right) \cdot S_{TP} \qquad \text{(S8a)}$$

where

$$S_{TP} = 60 \cdot \left(1 + e^{\frac{E_c - E_f}{kT}}\right) \cdot ln\left(1 + e^{\frac{E_f - E_c}{kT}}\right)$$

In FinFETs, as have been discussed in the main manuscript and **Supplementary Note 4**, $C_{div,<Vth} \rightarrow 0$, thus $C_{div}=C_Q+C_{div,<Vth} \rightarrow C_Q$, $C_{MOS}=C_{OX} \cdot C_{div}/(C_{OX}+C_{div}) \rightarrow C_{OX} \cdot C_Q/(C_{OX}+C_Q)$,

$$S_{NC-FET} = \left(1 + \frac{C_Q}{C_{OX}}\right) \cdot \left(1 - \frac{C_{MOS} + C_{frin,s/d-IMG}}{|C_{NC}|}\right) \cdot S_{TP}$$

$$= \frac{C_Q}{C_{MOS}} \cdot \left(1 - \frac{C_{MOS}+C_{frin,s/d-IMG}}{|C_{NC}|}\right) \cdot S_{TP} \qquad \text{(S8b)}$$

In the above- threshold regime, $C_Q >> C_{OX}$, $C_{MOS} \rightarrow C_{OX}$,

$$S_{NC-FET} \rightarrow \frac{C_Q}{C_{OX}} \cdot \left(1 - \frac{C_{OX} + C_{frin,s/d-IMG}}{|C_{NC}|}\right) \cdot S_{TP}$$

In the subthreshold regime, $C_Q \rightarrow 0$, $C_{MOS} \rightarrow 0$, $C_Q/C_{MOS}=(C_{OX}+C_Q)/C_{OX} \rightarrow 1$. **Equation S8b** is reduced to

$$SS_{NC-FET} = \left(1 - \frac{C_{frin,s/d-IMG}}{|C_{NC}|}\right) \cdot 60 \qquad \text{(S8c)}$$

The derived S/S_{TP} and $SS/60$ formula are plotted in **Supplementary Figure 6c**. The minimum SS (SS_{min}) that does not suffer from hysteresis can be obtained at $|C_{NC}|=C_{OX}+C_{frin,s/d\text{-}IMG}$, at which **Equation S8c** becomes

$$SS_{min} = \left(1 - \frac{C_{frin,s/d-IMG}}{C_{OX} + C_{frin,s/d-IMG}}\right) \cdot 60$$

$$= \left(\frac{C_{OX}}{C_{OX} + C_{frin,s/d-IMG}}\right) \cdot 60$$

Interestingly, the NC design space (between 0 and 1/(C_{OX}+$C_{frin,s/d\text{-}IMG}$)) and SS_{min} become irrelevant w.r.t. C_Q, and only dependent on C_{OX} and $C_{frin,s/d\text{-}IMG}$. This is because $C_{frin,s/d\text{-}IMG}$ provide parasitic charge in both subthreshold and above-threshold regime to induce polarization in NC, which unlocks the dependence of polarization on mobile charge (or C_Q) in "good" FETs. As indicated by the SS_{min} formula, SS_{min} can be effectively reduced by increasing the ratio of $C_{frin,s/d\text{-}IMG}$ to C_{OX}, which perfectly explains the previous report that short-channel NC FinFETs with IMG can achieve small *SS*. For long-channel NC-FETs, large overlap between source/drain and IMG, w.r.t. channel length, can be designed to reduce *SS*, which is confirmed by simulation of a Si SOI NC-FET, as shown in **Supplementary Figure 6d**.

Supplementary Note 9: A Rule for Polarization-Dynamics-Based Interpretation of the Subthermionic *SS*

As proved above and in the main text, it is very difficult to obtain steep slope in quasi-static condition from those NC-FETs made on "good" FET platforms without the help of IMG. There have been many studies that interpret the observed subthermionic *SS* in various reported "NC-FETs" as the transient effects during measurement[15,16] and/or intricate FE polarization dynamics $P(E, t)$[17-19]. Because of the great complexity, there hasn't been any consensus so far. Here, we point out a constraint or rule that these interpretations must obey, in order to correctly capture the observed subthermionic *SS*.

Ever since the proposal of NC-FETs[2], an approximation, $Q_{MOS} = D_{FE} = \varepsilon_0 E_{FE} + P_{FE} \approx P_{FE}$, has been widely used to describe the relation between the polarization in the FE material and charge in the MOSFET underneath. This approximation is obviously inconsistent in physics as pointed out by Liu et al.,[20] but remains mathematically valid in most FE memories and FE-DE double-layer capacitors, as well as in NC-FETs in the turn-on condition. On one hand, in those conditions, sufficient charge from the electrode plates or the channel of the MOSFET underneath can be supplied to compensate/induce strong polarization in the FE layer. On the other hand, for most FEs, $|\varepsilon_{FE}| >> \varepsilon_0$, thus $P_{FE} >> \varepsilon_0 E_{FE}$, and $Q_{MOS} \approx P_{FE}$. However, this approximation can be misleading in the subthreshold regime of NC-FETs. As discussed above, Q_{MOS} is negligibly small in the subthreshold regime of NC-FETs that are made on a

“good” FET platform and if an IMG is not employed, i.e.,

$$Q_{MOS} = D_{FE} = \varepsilon_0 E_{FE}(t) + P_{FE}(t) \rightarrow 0 \qquad \text{(S9)}$$

where t is the additional time dimension that determines the FE polarization. Note that this equation stays valid in any transient process. In this condition, the approximation of $Q_{MOS} \approx P_{FE}$ will result in a trivial solution, i.e., $Q_{MOS} \approx E_{FE} \approx P_{FE} \rightarrow 0$. In fact, **Equation S9** describes a non-conventional self-sustaining polarization scheme, in the absence of compensating charge for FE polarization. Simply speaking, the polarization in the FE can be sustained by the development of a non-trivial electric field E_{FE}, instead of relying on the compensating charge from external circuitry. As shown in the **Supplementary Figure 7a**, the green and blue dotted curves represent the quasi-static or stable P-E traces based on NC theory and conventional FE polarization switching theory, respectively, and **Equation S9** is essentially a self-sustaining line with a small ($|\varepsilon_{FE}| >> \varepsilon_0$) and negative slope, which translates to two pieces of important physics. On one hand, the FE polarization in the subthreshold regime is weak, most likely a result of significant depolarization. On the other hand, the residual weak polarization can be exploited to build up a negative electric field in the FE, and hence a negative V_{FE}, (**Supplementary Figure 7b**) in the subthreshold regime, as long as the polarization dynamics $P(E, t)$ allow the transient P-E operation point to evolve (with t and V_g) on the self-sustaining line and away from the origin (grey circle), as shown in **Supplementary Figure 7a**. This finding sets a rule for the future efforts in exploring potential polarization dynamics to capture the subthermionic *SS*s of various reported “NC-FETs”.

Supplementary Figure 1

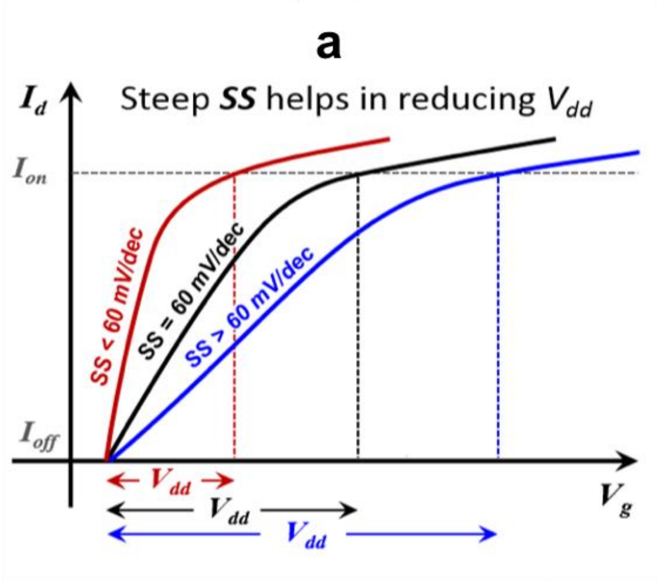

b

$$SS = \left(\frac{d[logI_d]}{dV_g}\right)^{-1} = \frac{dV_g}{d\varphi_{ch}} \cdot \left(\frac{d[logI_d]}{d\varphi_{ch}}\right)^{-1}$$

Electrostatics — Transport

	NEMS	NC FET	Mott FET	BTBT FET	i-MOS	SupL FET	Feedback FET
**Pros**	Ultra-small *SS*, High I_{on}	Manufact-ure friendly, High I_{on}	Ultra-small *SS*	Small *SS*, Weak Temp. dependence	Ultra-small *SS*	High I_{on}	Manufacture friendly Ultra-small *SS*
**Cons**	Manufacture, Reliability, CMOS compatibility	Low operation frequency	Hysteresis, Low operation frequency	Low I_{on}	Large V_{ds}, Reliability	Manufact-ure, Scalability	Unscalable V_{ds} Hysteresis, Variability, Reliability

Supplementary Figure 1. The importance of steep slope in FETs and several representative steep-slope devices. a The advantage of small subthreshold swing (*SS*) in terms of saving supply voltage V_{dd}. **b** The formula of *SS*, along with several steep-slope device concepts and their pros and cons. NEMS is nano-electro-mechanical switch[1]; NC FET is negative capacitance FET[2]; Mott-G FET is phase change material gated FET[3]; BTBT FET is band-to-band tunneling FET[4]; i-MOS is impact ionization MOSFET[5]; SupL FET is Super-Lattice FET[6]; Feedback FET employs charge-barrier positive feedback to achieve steep slope[7].

Supplementary Figure 2

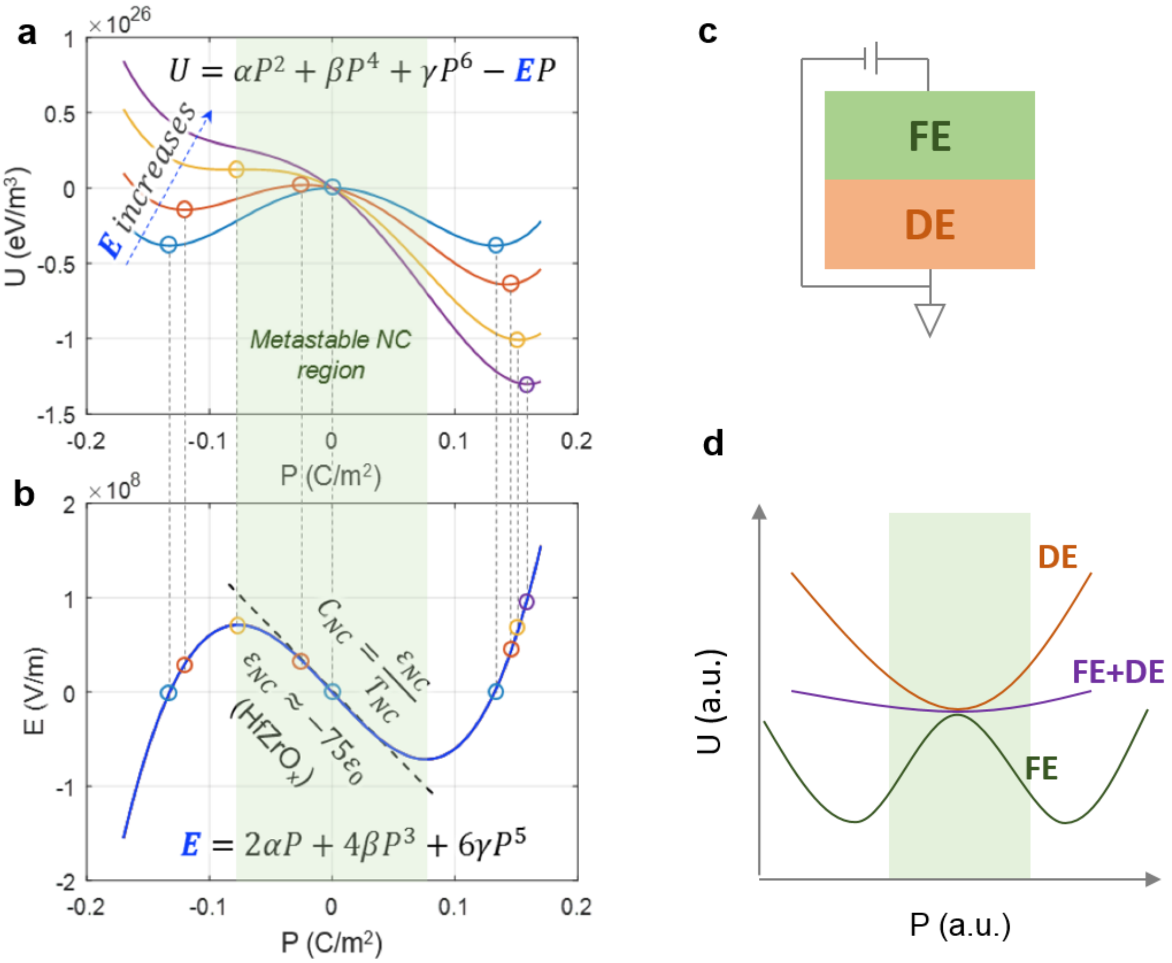

Supplementary Figure 2. Energy landscape of ferroelectric polarization. a Energy landscape (*U*) of a ferroelectric (FE) material in terms of polarization (*P*) and external electric field *E*. **b** Stable (including metastable) *P*-*E* relation derived from **a**. α=-7×10^{8} mF^{-1}, β=2×10^{10} m^{5}F^{-1}C^{-2}, γ=0 m^{9}F^{-1}C^{-4}. The shaded region represents the negative capacitance (NC) state. **c** Schematic of a FE - normal dielectric (DE) stack. **d** Schematic energy landscape of an individual FE, an individual DE, and a FE-DE stack (absolute value of the FE capacitance is made larger than the DE capacitance, i.e. $|C_{FE}| \geq C_{DE}$).

Supplementary Figure 3

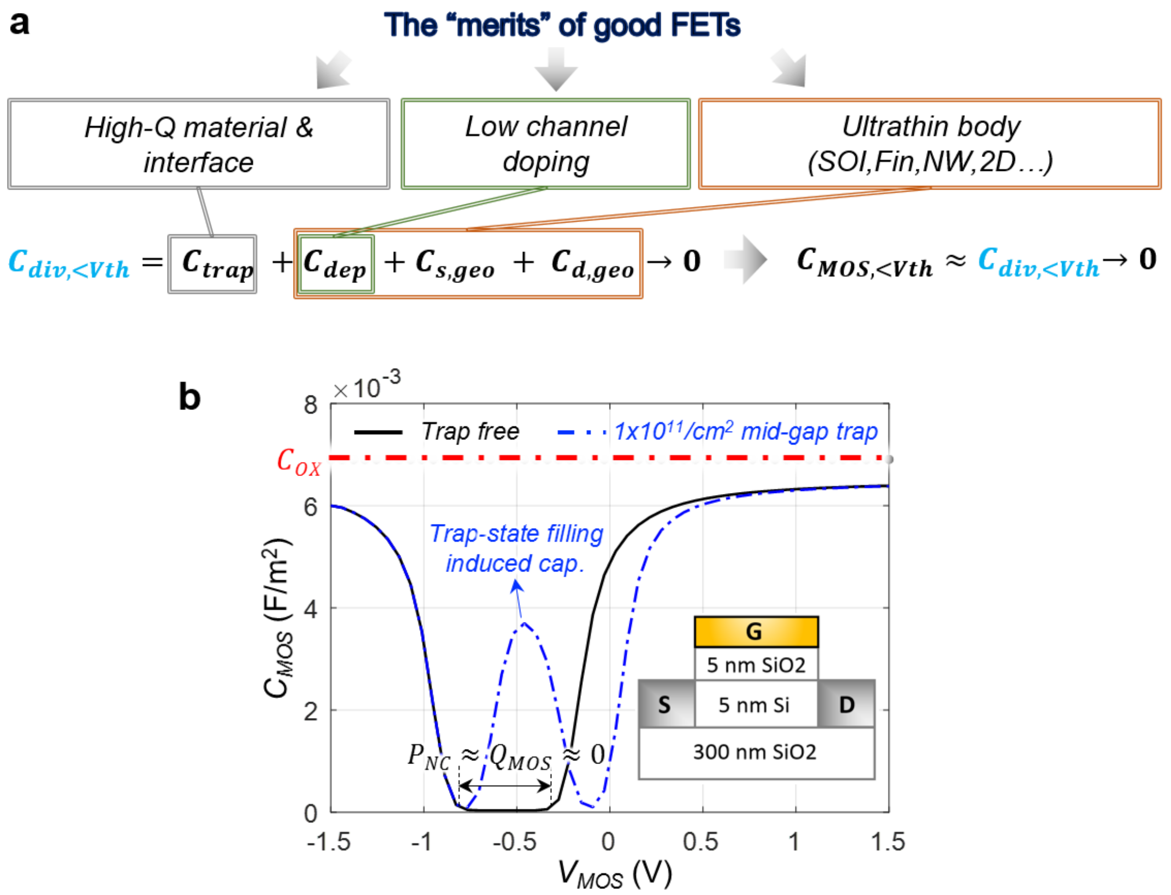

Supplementary Figure 3. The limitation of good FET platform for NC-FET construction. a Modern/good FETs have negligible $C_{div,<Vth}$, and hence $C_{MOS,<Vth}$, i.e., no NC polarization ($P_{NC} \approx Q_{MOS}$), or equivalently A_v benefit, in the subthreshold regime. **b** Simulated C_{MOS} in an ultrathin Si SOI MOSFET (see inset sketch) with and without traps. Note that trap states can introduce appreciable C_{trap}, and hence $C_{div,<Vth}$.

Supplementary Figure 4

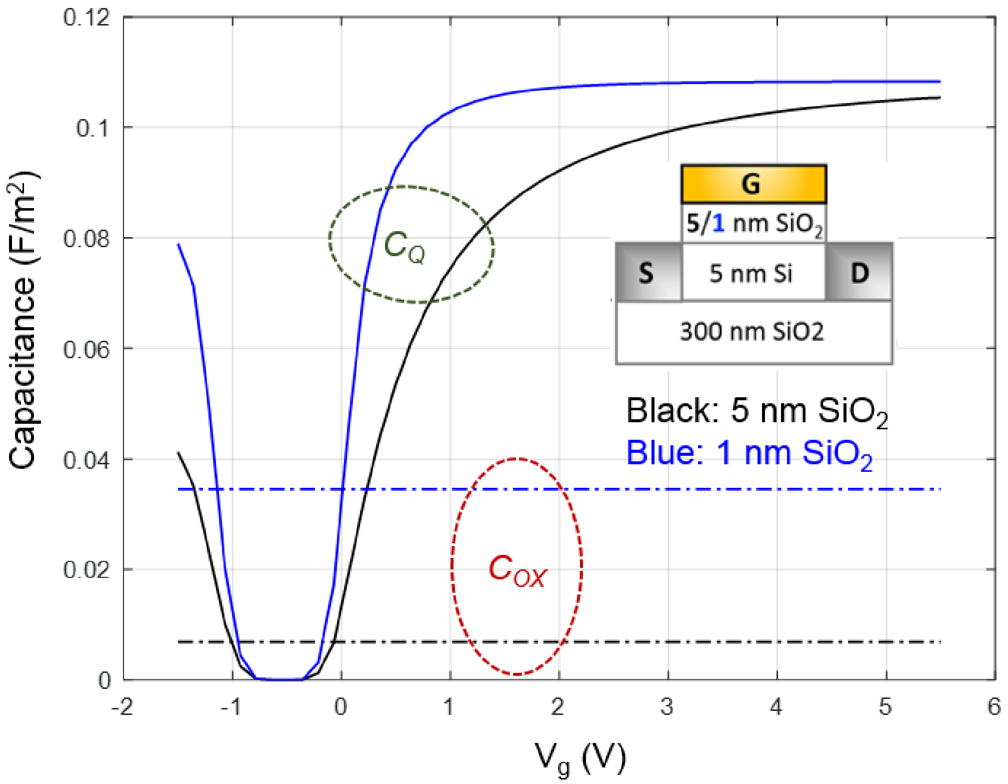

Supplementary Figure 4. The large quantum capacitance in FETs. Simulated C_Q in an ultrathin Si SOI MOSFET. Even in such a thin film, C_Q is still much larger w.r.t. the 1 nm SiO_2 gate oxide capacitance C_{OX}. Note that C_Q for 1 nm and 5 nm SiO_2 gate oxide will converge to the same value when V_g keeps increasing, i.e., the maximum C_Q is independent of gate oxide thickness.

Supplementary Figure 5

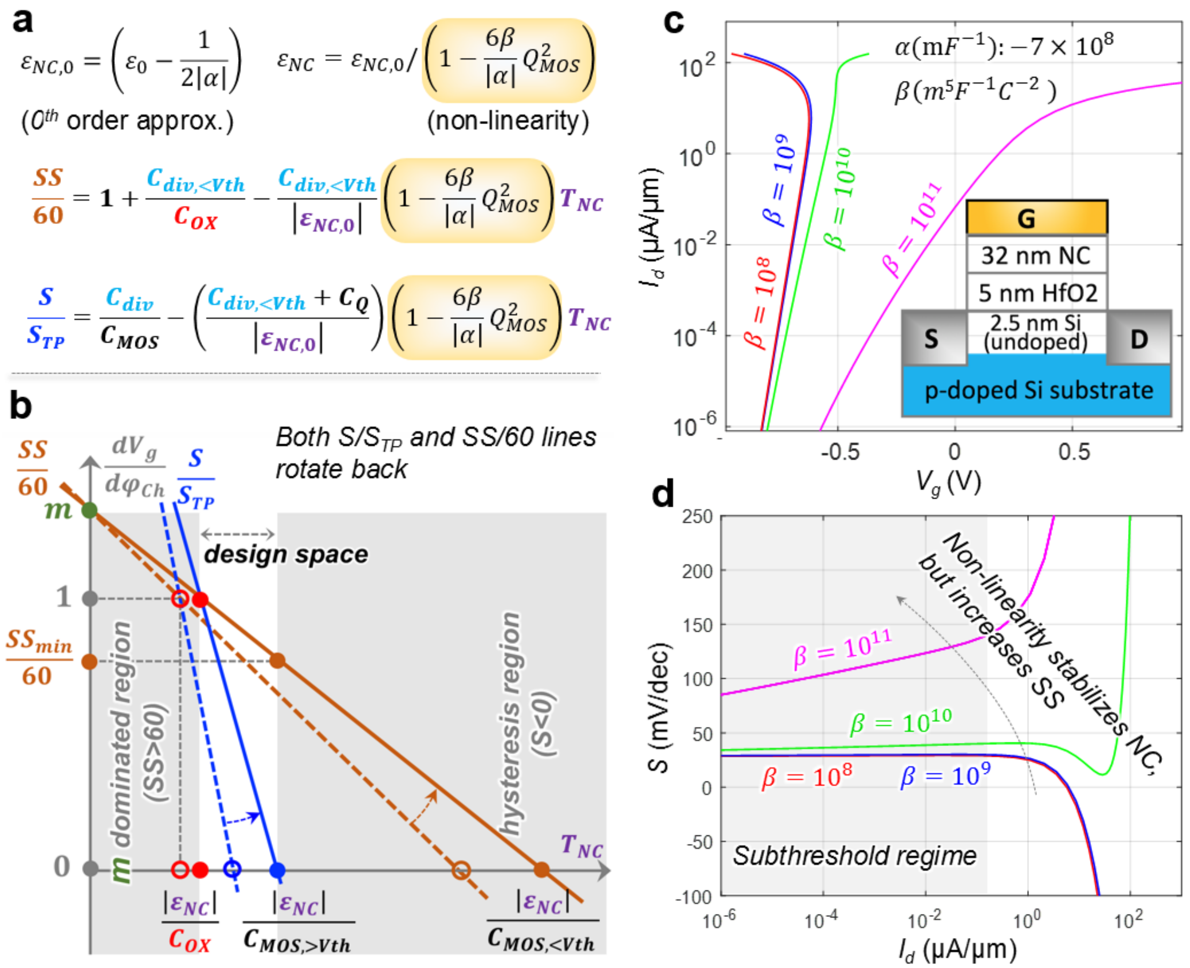

Supplementary Figure 5. Non-linearity of NC. a The non-linearity of NC dynamically increases $|\varepsilon_{NC}|$ (or $|C_{NC}|$) with the charge density on the MOSFET gate, Q_{MOS}, or gate bias. **b** It rotates back both *S/S*$_{TP}$ and *SS/60* lines, which enlarges the NC design space constrained by C_Q, but increases *SS*. **c**,**d** Numerical simulation of a NC-FET (inset) shows that with stronger NC non-linearity, i.e., larger β, the hysteresis is suppressed, but *SS* gets degraded, which is consistent with the uncovered NC non-linearity effects in **a** and **b**.

Supplementary Figure 6

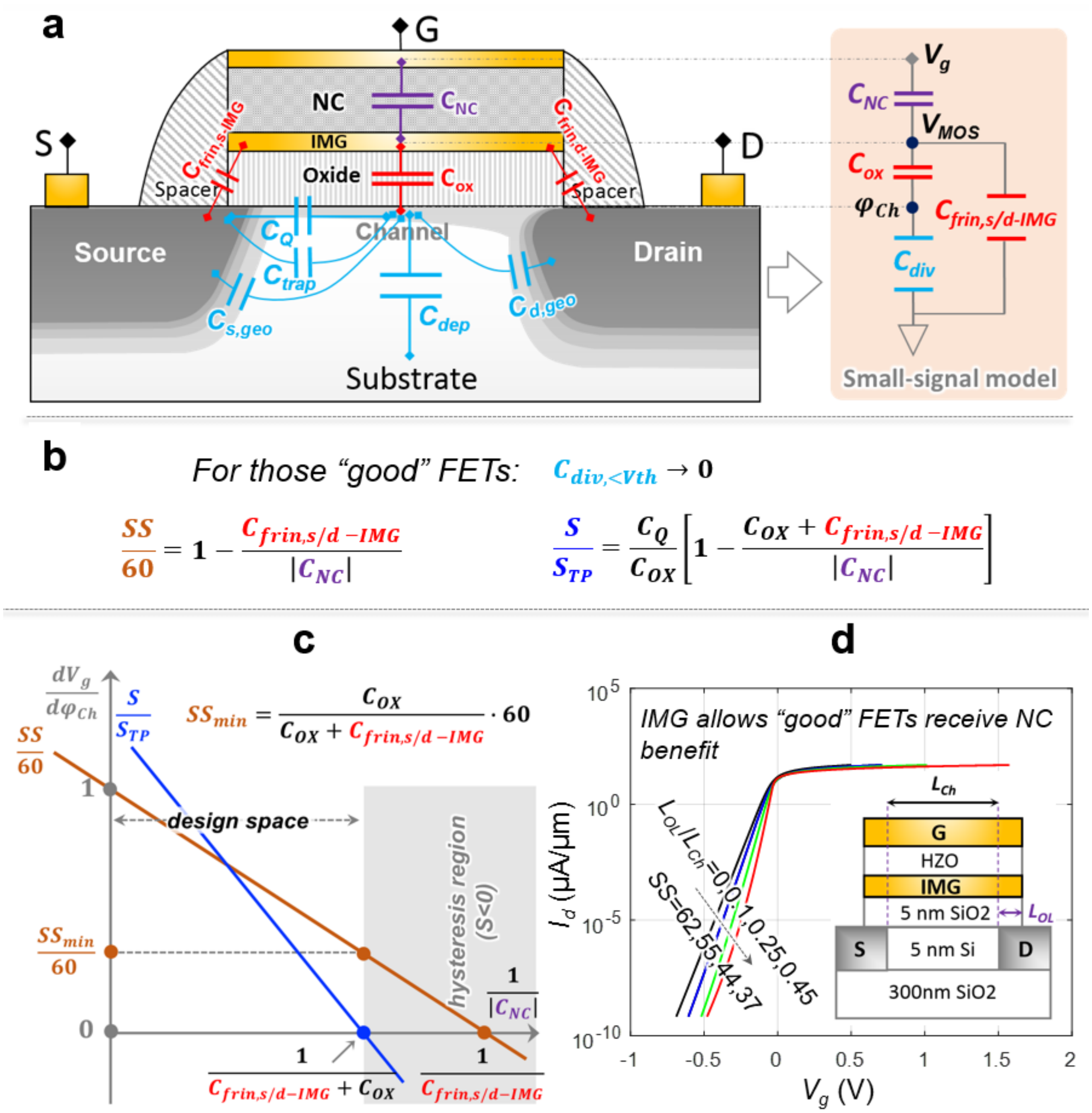

Supplementary Figure 6. The physics of internal metal gate based NC-FETs. a Capacitors in a NC-FET with internal metal gate (IMG). **b** Revisited S and SS formula. **c** IMG can borrow charge from fringing/overlap capacitance in subthreshold regime, thereby unlocking the polarization from C_Q, and enlarging the NC design space for small SS_{min}. **d** Simulation results based on a Si SOI MOSFET verify the uncovered IMG mechanism. The larger the L_{OL}/L_{Ch}, more the NC benefits.

Supplementary Figure 7

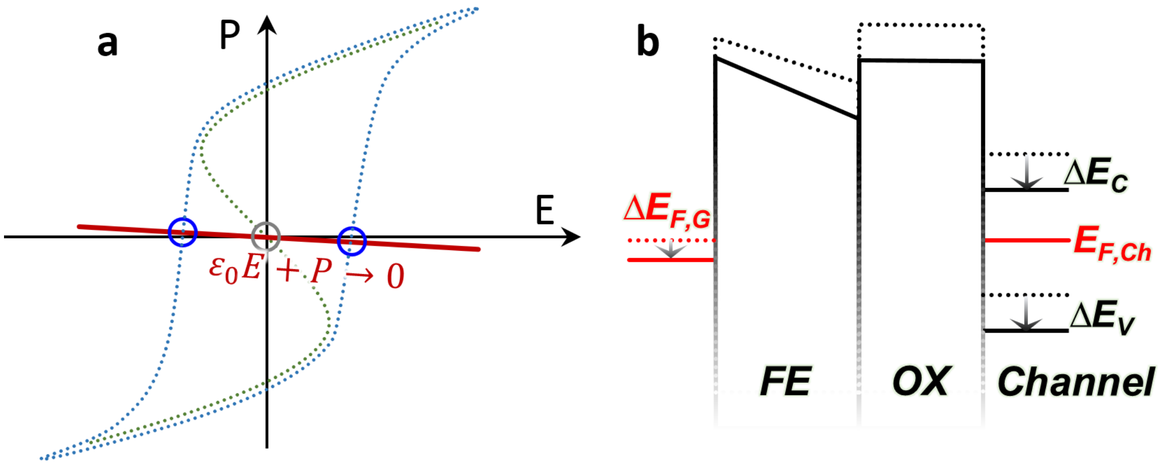

Supplementary Figure 7. Expected P-E relation in the subthreshold regime. a To receive NC benefit in the subthreshold regime, i.e., negative voltage on the FE layer, the transient FE operation points predicted by any polarization dynamics model should be along the self-sustaining line ($\varepsilon_0 E_{FE} + P_{FE} \to 0$) and away from the origin (grey circle). Dotted curves represent possible polarization traces. **b** Band diagrams of a NC-FET in the subthreshold regime before (dotted lines) and after (solid lines) a gate voltage tuning.

Supplementary Table 1

Ref.	FE/Channel	FET Structure	DC Hysteresis	AC Hysteresis	SS_{min} (@DC)	I_d (with SS<60)	I_{ON}/I_{OFF}
8	$Hf_xAl_{1-x}O$/Si	Bulk MOSFET	~ 4 mV	~ 0.7V @ 0.1µs pulse	~ 39 mV/dec	< 10 nA/µm	10^6
9	$Hf_xAl_{1-x}O$/Si	Bulk MOSFET	~ 20 mV	N/A	~ 24 mV/dec	< 1 nA/µm	10^8
10	$Hf_xAl_{1-x}O$/Si	FinFET	~ 29 mV	N/A	~ 55 mV/dec	~ 1 nA/µm	10^9
11	$Hf_xZr_{1-x}O$/Ge	FinFET	~ 17 mV	N/A	~ 43 mV/dec	< 1 nA/µm	10^5
12	$Hf_xZr_{1-x}O$ /MoS2	Bottom Gate	~ 10 mV	~ 0.2 V @ ~ ms pulse	52.3 mV/dec	< 1 nA/µm	~ 10^7

Supplementary Table 1: Collected data from several recently reported "NC-FETs". (N/A: Not Available)

ACKNOWLEDGEMENTS

The research outlined in this Supplementary Information file and the corresponding main article (https://doi.org/10.1038/s41467-019-13797-9) from the Nanoelectronics Research Lab at UCSB was supported by the ARO (grant W911NF1810366), JST CREST program (grant SB180064), and Intel Corporation.

Supplementary References

1. Ionescu, A. M. et al. Modeling and design of a low-voltage SOI suspended-gate MOSFET (SG-MOSFET) with a metal-over-gate architecture. *Proc. ISQED*, 496-501 (2002).
2. Salahuddin, S. & Datta, S. Use of negative capacitance to provide voltage amplification for low power nanoscale devices. *Nano Lett*. **8**, 405–410 (2008).
3. Huang, Q., Huang, R., Pan, Y., Tan, S. & Wang, Y. Resistive-gate field-effect transistor: A novel steep-slope device based on a metal-insulator-metal-oxide gate stack. *IEEE Elec. Dev. Lett*. **35**, 877-879 (2014).
4. Baba, T. Proposal for surface tunnel transistors. *Jpn. J. Appl. Phys*. **31**, 455–457 (1992).
5. Gopalakrishnan, K., Griffin, P. & Plummer, J. Impact ionization MOS (I-MOS)-Part I: Device and circuit simulations. *IEEE Tran. Elec. Dev.* **52**, 69-76 (2005).
6. Gnani, E., Reggiani, S., Gnudi, A. & Baccarani, G. Steep-slope nanowire FET with a superlattice in the source extension. *Solid-State Electronics*. 108-113 (2011).
7. Padilla, A., Wing, Y., Shin, C., Hu, C. & King, L. Feedback FET: A novel transistor exhibiting steep switching behavior at low bias voltages. *IEEE Int. Elec. Dev. Meeting*. 1-4 (2008).
8. Lee, M. et al. Ferroelectric Al: HfO_2 negative capacitance FETs," *IEEE Int. Elec. Dev. Meeting*. 565-568 (2017).
9. Fan, C. et al. Energy-efficient HfAlOx NCFET: Using gate strain and defect passivation to realize nearly hysteresis- free sub-25mV/dec switch with ultralow leakage. *IEEE Int. Elec. Dev. Meeting*. 561-564 (2017).
10. Chung, W. et al. Hysteresis-free negative capacitance germanium CMOS FinFETs with bi-directional sub-60 mV/dec. *IEEE Int. Elec. Dev. Meeting*. 365-368 (2017).
11. Krivokapic, Z. et al. 14 nm ferroelectric FinFET technology with steep subthreshold slope for ultra low power applications. *IEEE Int. Elec. Dev. Meeting*. 357–360 (2017).
12. Si, M. et al. Steep-slope hysteresis-free negative capacitance MoS_2 transistors. *Nature Nano*. **13**, 24–28 (2018).
13. Jain, A. & Alam, M. Stability constraints define the minimum subthreshold swing of a negative capacitance field-effect transistor. *IEEE Trans. Electron Devices*. **61**, 2235–2242 (2014).
14. Ota, H. et al. Fully coupled 3-D device simulation of negative capacitance FinFETs for sub 10 nm integration. *IEEE Int. Elec. Dev. Meeting*. 318–321 (2016).
15. Saha, A., Datta, S. & Gupta, S. "Negative capacitance" in resistor-ferroelectric and ferroelectric-dielectric networks: Apparent or intrinsic? *J. Appl. Phys*. **123**, 105102-1 (2018).

16. Kobayashi, M., Ueyama, N., Jang, K. & Hiramoto, T. Experimental study on polarization-limited operation speed of negative capacitance FET with ferroelectric HfO_2. *IEEE Int. Elec. Dev. Meeting*. 314–317 (2016).
17. Wang, H. et al. New insights into the physical origin of negative capacitance and hysteresis in NCFETs. *IEEE Int. Elec. Dev. Meeting.* 31.1.1–31.1.4 (2018).
18. Li, X. & Toriumi, A. Direct relationship between sub-60 mV/dec subthreshold swing and internal potential instability in MOSFET externally connected to ferroelectric capacitor. *IEEE Int. Elec. Dev. Meeting*. 31.3.1–31.3.4 (2018).
19. Jin, C., Jang, K., Saraya, T., Hiramoto, T. & Kobayashi, M. Experimental study on the role of polarization switching in subthreshold characteristics of HfO_2-based ferroelectric and anti-ferroelectric FET. *IEEE Int. Elec. Dev. Meeting*. 31.5.1–31.5.4 (2018).
20. Liu, Z., Bhuiyan, M., & Ma, T., A critical examination of 'quasi-static negative capacitance' (QSNC) theory. *IEEE Int. Elec. Dev. Meeting*. 31.2.1–31.2.4 (2018).
